# BALANCING WORK AND HEALTH IN AGING: EMPLOYMENT AND ARTHRITIS DEVELOPMENT AMONG MIDLIFE AND OLDER WOMEN

**DOI:** 10.1093/geroni/igae098.2372

**Published:** 2024-12-31

**Authors:** Micah Tan, Paulin Straughan

**Affiliations:** Princeton University, Princeton, New Jersey, United States; Singapore Management University, Singapore, Singapore

## Abstract

With population aging, many countries have opted to delay mandatory retirement ages and to encourage workers to continue working into mid- and late-life. However, the implications of such policies for the health of older workers are still understudied. Existing research has demonstrated that women tend to experience greater negative effects on their physical health from working due to having to also shoulder a greater responsibility for household chores and caregiving. Yet, there is a paucity of research examining if such effects are present for older workers, as well as in East-Asian contexts where additional responsibilities at home are greater for women. As such, utilizing two waves of data from the Singapore Life Panel collected in January 2018 and January 2023, the current research aims to understand the physical health effects of work among middle-aged and older-adult women relative to men in Singapore. With a final sample size of 4225 respondents (mean age at baseline = 60.38 years) that had not yet been diagnosed with arthritis at baseline and in fully-adjusted models controlling for marital status, having children, and levels of social support available, we find that women who were working at baseline had a greater likelihood of being diagnosed with arthritis at follow-up relative to women who were not working. In comparison, working at baseline is not found to have any effect on the likelihood of being diagnosed with arthritis among men in the sample. The implications of these findings for health and labor policies in aging populations are discussed.

